# A deep-learning estimate of the decadal trends in the Southern Ocean carbon storage

**DOI:** 10.1038/s41467-022-31560-5

**Published:** 2022-07-13

**Authors:** Varvara E. Zemskova, Tai-Long He, Zirui Wan, Nicolas Grisouard

**Affiliations:** Department of Physics, University of Toronto, Toronto, ON Canada

**Keywords:** Physical oceanography, Carbon cycle, Marine chemistry, Physical oceanography, Climate and Earth system modelling

## Abstract

Uptake of atmospheric carbon by the ocean, especially at high latitudes, plays an important role in offsetting anthropogenic emissions. At the surface of the Southern Ocean south of 30^∘^S, the ocean carbon uptake, which had been weakening in 1990s, strengthened in the 2000s. However, sparseness of in-situ measurements in the ocean interior make it difficult to compute changes in carbon storage below the surface. Here we develop a machine-learning model, which can estimate concentrations of dissolved inorganic carbon (DIC) in the Southern Ocean up to 4 km depth only using data available at the ocean surface. Our model is fast and computationally inexpensive. We apply it to calculate trends in DIC concentrations over the past three decades and find that DIC decreased in the 1990s and 2000s, but has increased, in particular in the upper ocean since the 2010s. However, the particular circulation dynamics that drove these changes may have differed across zonal sectors of the Southern Ocean. While the near-surface decrease in DIC concentrations would enhance atmospheric CO_2_ uptake continuing the previously-found trends, weakened connectivity between surface and deep layers and build-up of DIC in deep waters could reduce the ocean’s carbon storage potential.

## Abstract (web summary)

Dissolved carbon concentrations in the ocean interior are computed by a deep-learning model using ocean surface data. In the Southern Ocean, they decreased in the 1990s-2000s and increased since 2010, reducing anthropogenic carbon uptake potential.

## Introduction

Atmospheric CO_2_ concentrations have been rising since the preindustrial era, in large part due to burning of fossil fuels and land-use changes, such as deforestation and urbanization^1,2^. Global carbon budget models estimate that oceans absorb about 25% of anthropogenic carbon emissions^3^. Polar regions play a particularly important role in carbon uptake, i.e., the transfer of CO_2_ from air into the ocean. Indeed, carbon uptake increases with decreasing temperature and increasing wind speed, which enhances gas solubility and mixing, respectively at the surface^4^. Consequently, it is estimated that the Southern Ocean is responsible for approximately 40% of the oceanic carbon sink of the anthropogenic emissions^5^, where persistent zonal winds are strong and temperatures are relatively cold. Biological uptake of carbon has also been shown to play an important role in the Southern Ocean^6–8^. Biological uptake predominantly occurs in the spring and summer, importantly when CO_2_ solubility is weak, and previous studies^6^ have found decadal variability in primary production, which subsequently affects the ocean carbon sink.

There has been concern regarding a declining trend in the Southern Ocean carbon uptake from the 1980s into early 2000s^9,10^. However, recent multidecadal analysis of surface ocean CO_2_ measurements found a reversed trend, i.e., that the ocean carbon uptake has been increasing in the 2000s, attributed to changes in ocean circulation, which are primarily due to non-trivial shifts in wind forcing^11^. However, carbon needs to be exported from the surface down into the ocean interior, where it cannot further exchange with the atmosphere^12^. The changes in this export are important not only for the climate but also marine chemistry. An increase in dissolved carbon has led to ocean acidification that subsequently affects marine organisms^13^. However, trends in carbon concentrations in the ocean interior are still poorly understood, primarily for two reasons. First, it is difficult to model biogeochemical cycles in ocean models^14^ and second, ocean measurements are spatially and temporally sparse^15,16^.

To address this sparseness of observations, we developed a deep-learning model^17^ that predicts concentrations of dissolved inorganic carbon (DIC) in the upper 4 km in the ocean using surface and near-surface variables: sea surface temperature, flow velocity at the surface, sea surface height, near-surface wind velocity, and surface CO_2_ partial pressure (pCO_2_). All of the input parameters are readily available via satellite measurements, with the exception of pCO_2_, which has been previously estimated by another neural network^18^ trained and tested with observational data from Surface Ocean CO_2_ Atlas (SOCAT).

We train our model in two phases (see Methods): first is the Biogeochemical Southern Ocean State Estimate (B-SOSE), which is a data assimilating ocean circulation model^14^. It is available at a high spatial and temporal resolution of 1/3^∘^ and 3-day resolution, respectively, and therefore provides a large volume of data for the initial training, especially in the deep layers, where fewer observational measurements are available. In the second phase, we use DIC measurements from Global Ocean Data Analysis Project version 2 (GLODAPv2) shipboard measurements (available at least up to 4 km depth)^19,20^ and Southern Ocean Carbon and Climate Observations and Modeling (SOCCOM) biogeochemical Argo floats (available up to 2 km depth)^21^. These measurements are used to correct any biases originating from the B-SOSE model used in the first phase. Similar to previous works on modeling pCO_2_^22^, we find that the model relative error is reduced when using a combination of shipboard and float measurements in the training set.

## Results

**Fig. 1:**
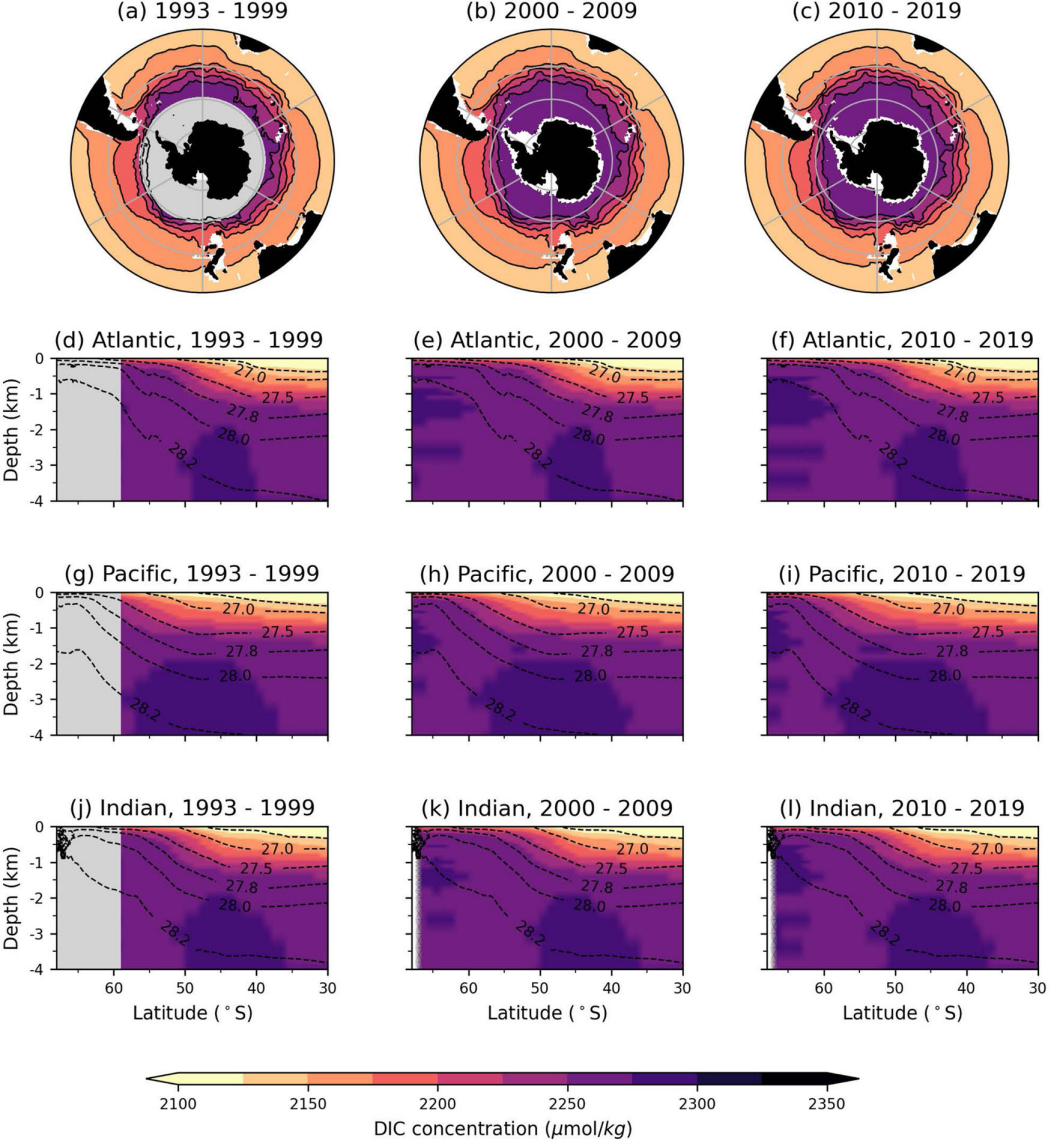
Dissolved inorganic carbon (DIC) concentrations computed using our deep learning model. **a**, **d**, **g**, **j** 1993–1999, (**b**, **e**, **h**, **k**) 2000–2009, (**c**, **f**, **i**, **l**) 2010-2019. (a–c) Decadal averages of DIC concentrations over top 1 km with contours, zonal means of (**d**–**f**) Atlantic, (**g**–**i**) Pacific, and (**j**–**l**) Indian Oceans. Black dashed contours correspond to isosurfaces of neutral density *γ*_*N*_ from B-SOSE averaged zonally and temporally over 2008–2012 (unlabeled contour: *γ*_*N*_ = 26.6 kg/m^3^). **a**–**c** were made with Natural Earth. Free vector and raster map data (naturalearthdata.com) using Cartopy^77^.

Using this deep-learning model, we computed the distribution of five-day-averaged DIC concentrations over the 1993−2019 period south of 30^∘^S^23^. The depth- and zonally-averaged DIC concentrations, separated into three ocean basins (Atlantic, Pacific, and Indian), are shown in Fig. 1 and averaged over three periods (1993–1999, 2000–2009, 2010–2019). As there are several climate variabilities that drive the Southern Ocean dynamics on time scales of years to decades, we align our temporal periods with previous studies following the changes in global observation system^9,11^ rather than any specific climatological cycle. Near the surface, DIC concentrations increase polewards with latitude and largely follow the neutral density surfaces in the interior, consistent with previous estimates^24^. The Pacific and Indian basins, which have older, bottom-sourced waters^25^ have higher DIC concentrations compared with the Atlantic basin, whose deep waters are ventilated more frequently^25^.

### Strengthening carbon sink in the 1990s

**Fig. 2:**
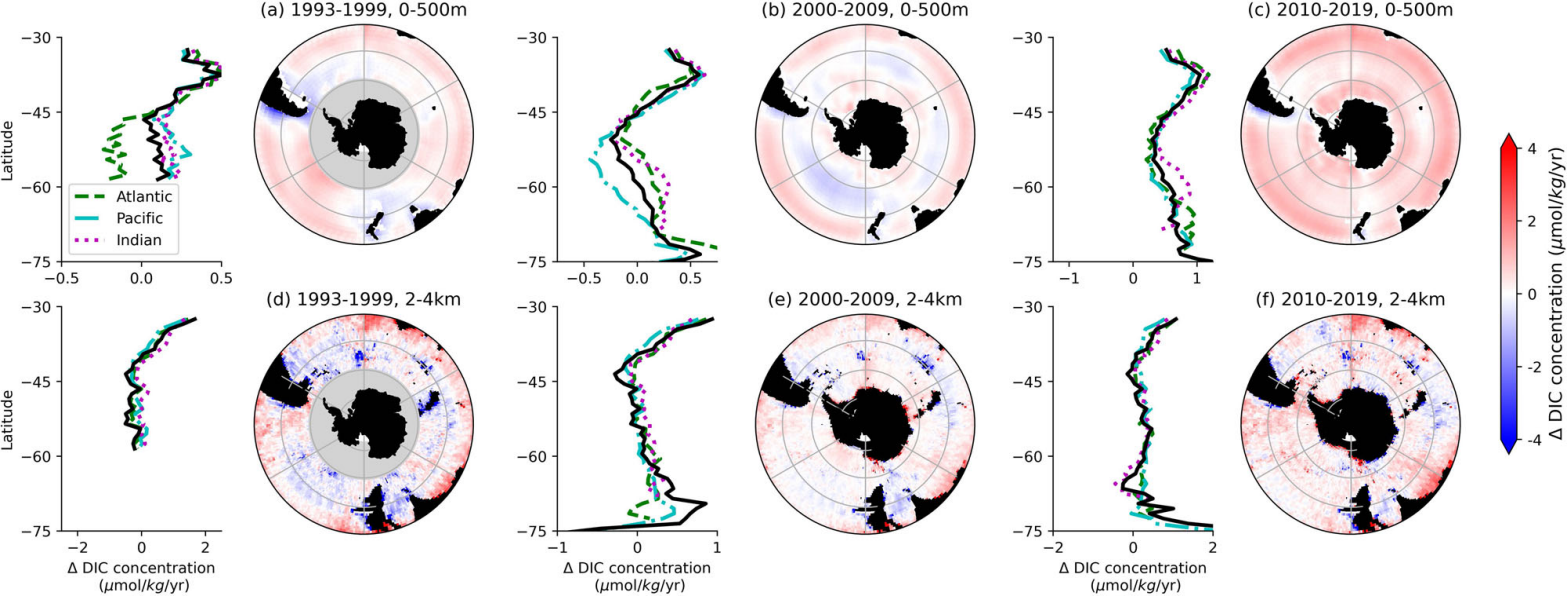
Linear trends in dissolved inorganic carbon (DIC) concentration. **a**–**c** averaged over top 500 m, (**d**–**f**) averaged over 2−4 km depth. Values are calculated over: (**a**, **d**) 1993–1999, (**b**, **e**) 2000-2009, and (**c**, **f**) 2010–2019. Linear trends outside the 5% significance level (*p* ≥ 0.05) are excluded. Areas shaded in gray indicate regions of insufficient data for trend calculations. Panels to the left of each colored trend plots show zonal averages for the entire Southern Ocean (black solid line), and the Atlantic (dashed green line), Pacific (dash-dot cyan line), and Indian sectors (dotted magenta line). Figures were made with Natural Earth. Free vector and raster map data (naturalearthdata.com) using Cartopy^77^.

**Fig. 3:**
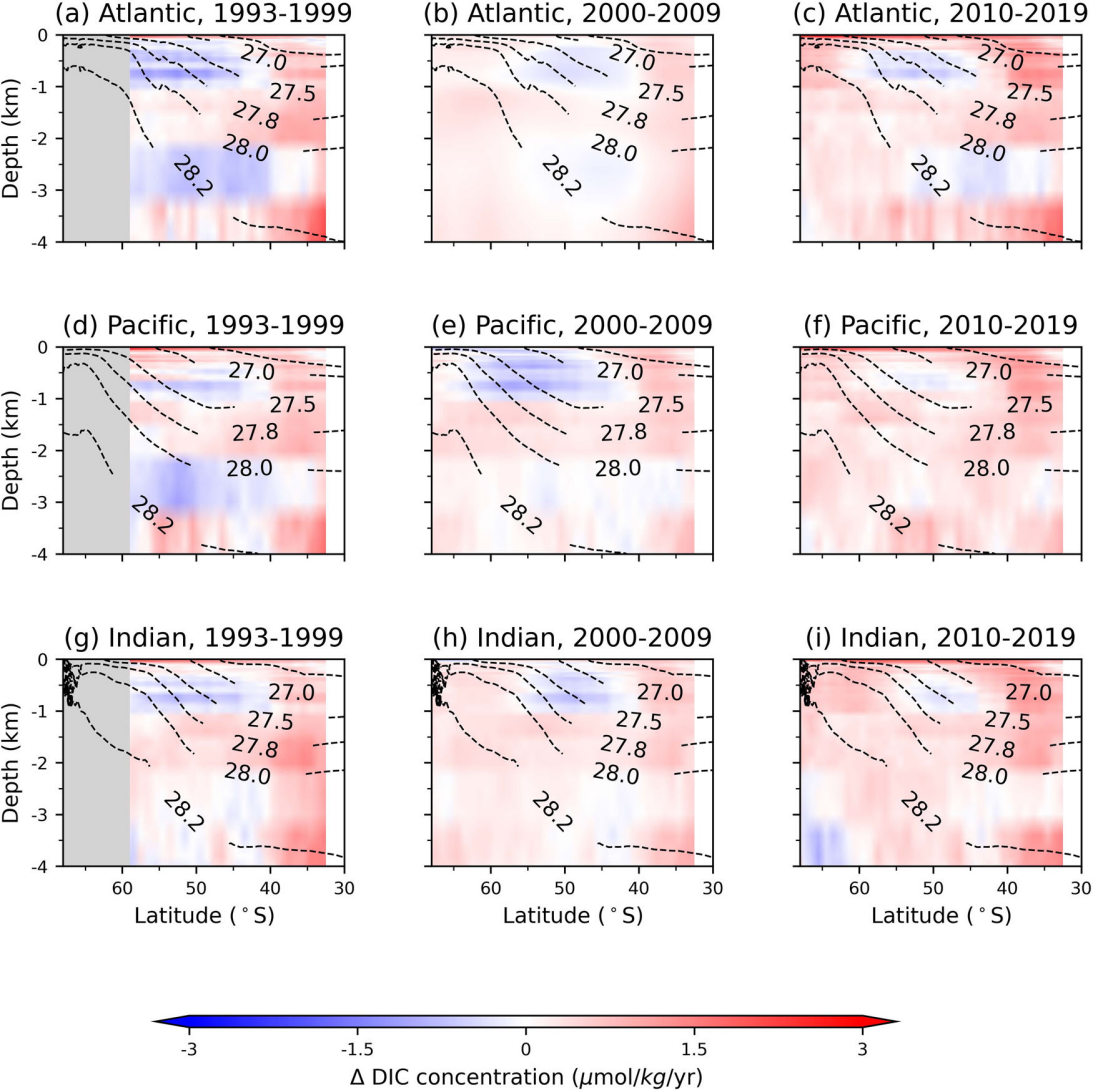
Linear trends in dissolved inorganic carbon (DIC) concentration with depth. **a**, **d**, **g** 1993–1999, (**b**, **e**, **h**) 2000–2009, (**c**, **f**, **i**) 2010–2019. Zonal means of (**a**–**c**) Atlantic, (**d**–**f**) Pacific, and (**g**–**i**) Indian Oceans. Black dashed contours correspond to isosurfaces of neutral density *γ*_*N*_ from B-SOSE averaged zonally and temporally over 2008–2012 (unlabeled contour: *γ*_*N*_ = 26.6 kg/m^3^).

**Fig. 4:**
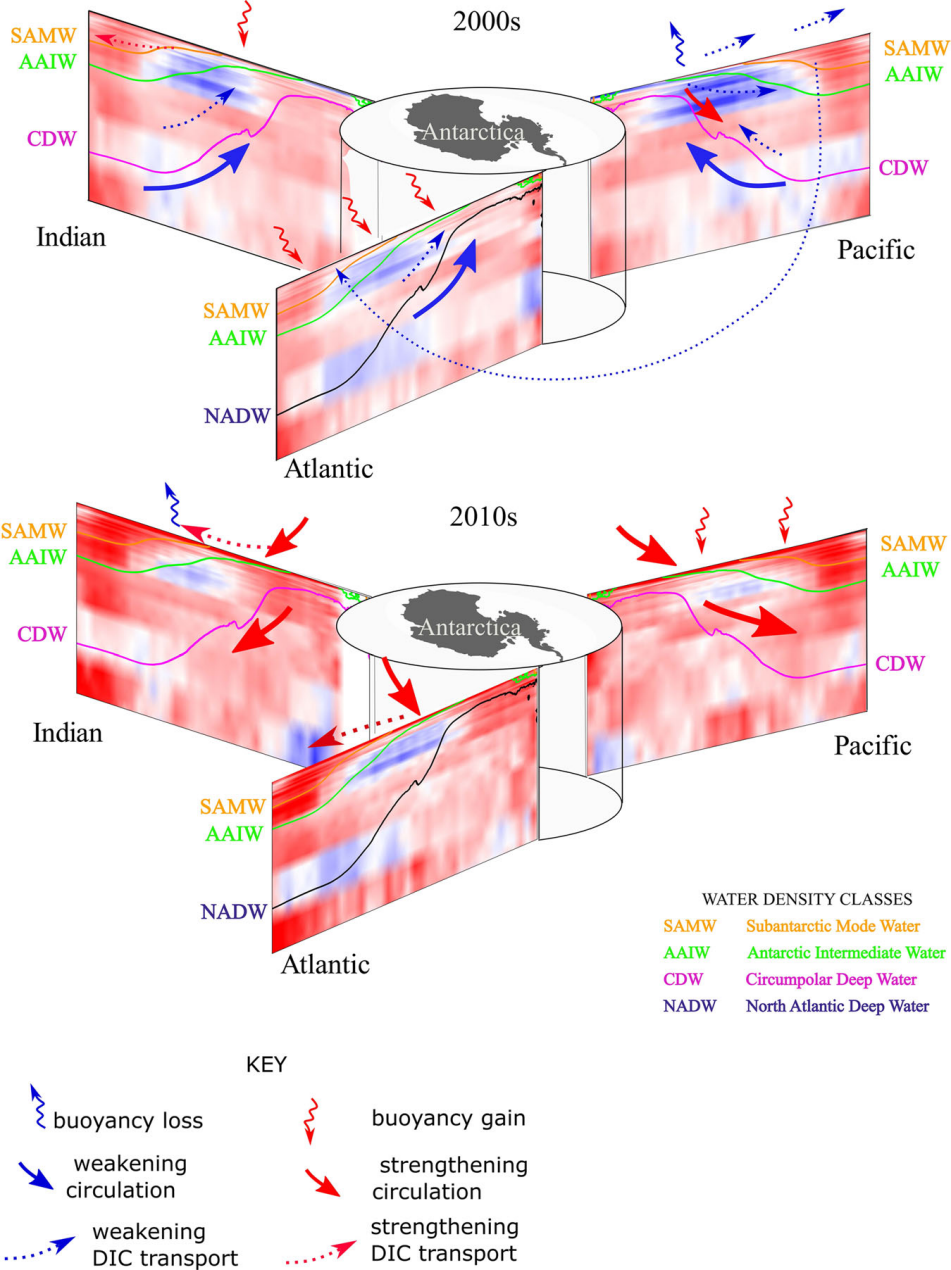
Schematic of the mechanisms affecting dissolved inorganic carbon (DIC) trends in the 2000s and 2010s between 30 and 75^∘^S broken down by ocean sectors. Solid colored lines trace out representative density surfaces of each water-mass: Subantarctic Mode Water (SAMW), Antarctic Intermediate Water (AAIW), Circumpolar Deep Water (CDW), North Atlantic Deep Water (NADW). Blue (red) color shading indicates decreasing (increasing) DIC trends. Curly arrows mark buoyancy forcing at the surface: blue (red) indicating buoyancy loss, i.e., input of denser water (buoyancy gain, i.e., input of lighter water). Solid thick arrows mark changes in ocean circulation: blue (red) indicating weakening (strengthening) flow in the indicated direction. Small dotted arrows mark relative strength of DIC transport: blue (red) indicating weakening (strengthening) transport or transport of lower (higher) DIC concentrations. Map of Antarctica was made with Natural Earth. Free vector and raster map data (naturalearthdata.com) using Cartopy^77^.

Between 1993 and 2009, DIC concentrations have decreased in the ocean interior, especially in the Pacific sector (Figs. 2 and 3 left and middle panels). The decreasing surface DIC trend, which subsequently lowers pCO_2_ at the ocean surface, is consistent with the previously found strengthening of the Southern Ocean carbon sink in the 2000s^11^. However, the changes in DIC concentrations are not zonally uniform, suggesting that distinct mechanisms may exist in different ocean basins (cf. Fig. 4 top). In the 2010s, DIC trends reversed, and DIC concentrations have been increasing, especially near the surface, possibly because the ocean surface was undersaturated and able to take up more carbon (cf. Figs. 2 and 3 right panels, Fig. 4 bottom).

**Fig. 5:**
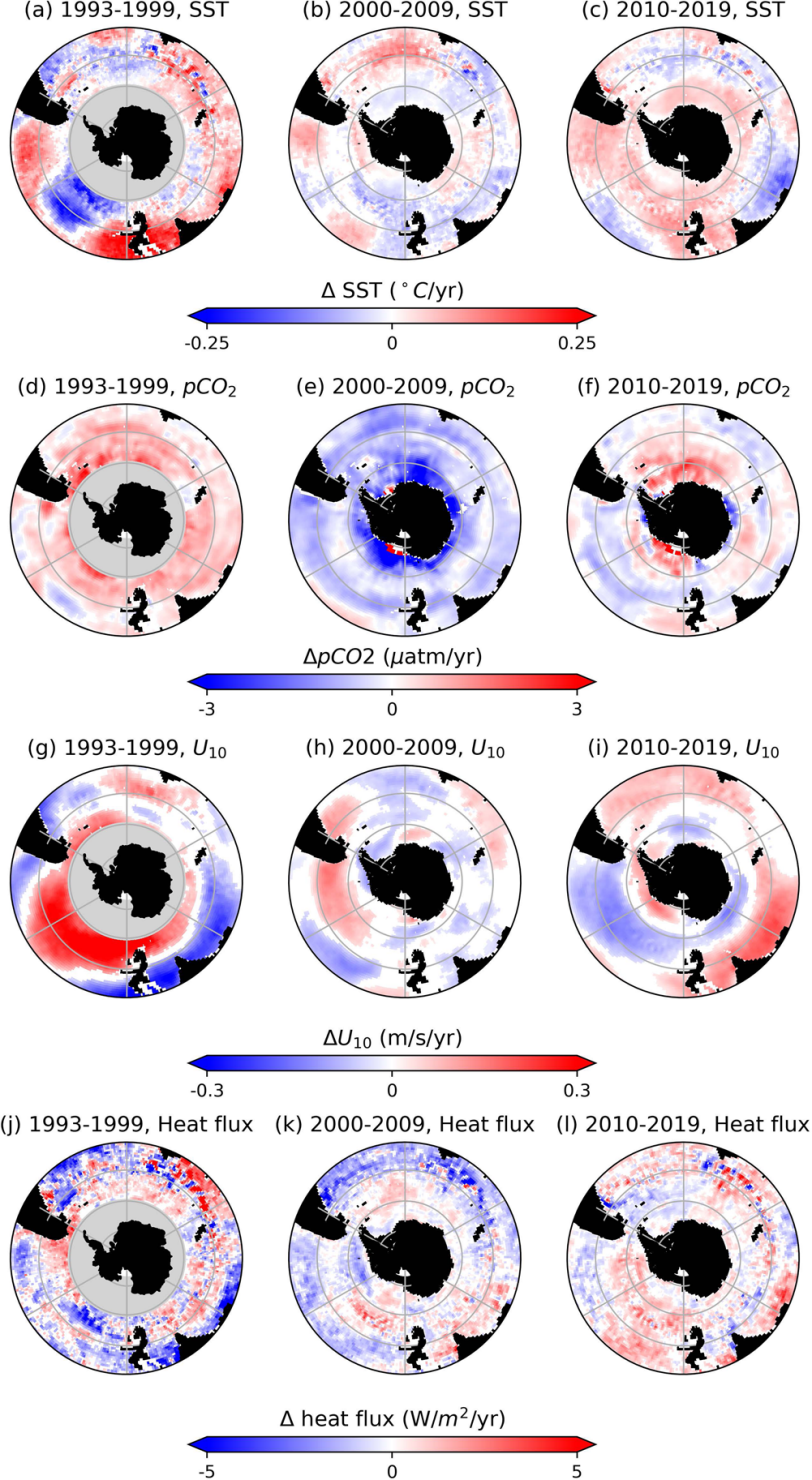
Annual trends of selected environmental variables used as inputs in the deep learning model. Annual trends for (**a**–**c**) sea surface temperature (SST), (**d**–**f**) difference between ocean and atmosphere *p*CO_2_, (**g**–**i**) near sea-surface zonal wind speed (*U*_10_), and (**j**–**l**) net sea surface heat flux. Trends are divided into three temporal periods: (**a**, **d**, **g**, **j**) 1993–1999, (**b**, **e**, **h**, **k**) 2000–2009, (**c**, **f**, **i**, **l**) 2010–2019. Satellite data sources for each of the environmental variables are given in “Methods”. Figures were made with Natural Earth. Free vector and raster map data (naturalearthdata.com) using Cartopy^77^.

In the 1990s, DIC mostly increased in the upper 1 km over the Pacific within the Antarctic Circumpolar region (50−60^∘^S; Figs. 2a and 3d). The predominantly positive phase of the Southern Annular Mode since the 1980s^26,27^ has been associated with the intensification and poleward shift in the Westerlies, the zonally persistent eastward winds at these latitudes (Fig. 5g). These stronger winds result in flow divergence near the surface and intensify upwelling of DIC-rich waters from the abyss^28^. Consistent with the signature of stronger upwelling, there is a decrease in DIC in deeper waters (Fig. 2d).

While there is also an increasing DIC trend in the South Atlantic and South Indian Oceans in the 1990s, in particular equatorward of 45^∘^S (Figs. 2a and 3a, g), the rates are lower than in the South Pacific. The zonal differences could be attributed to the zonal asymmetry in the atmospheric forcing^29^ that has resulted in greater intensification of the Westerlies over the Pacific than over the Atlantic or Indian sectors^11,30^ (cf. Fig. 5g). The overall increase in DIC is further consistent with the increase in sea surface pCO_2_ and increased outgassing or decreased uptake of atmospheric carbon by the Southern Ocean in response to the positive Southern Annular Mode^9,10,31^ (cf. Fig. 5d). Notably, the strong near-surface decrease in DIC in the Western Indian sector around 40−50^∘^S (cf. Fig. 2a) could be because of the increased stratification, which weakens the upwelling of carbon from the deep ocean, due to warming in this region over the previous several decades^32^ corresponding to increasing sea surface temperature in this region (cf. Fig. 5a).

### Zonal asymmetry in water-mass transformation and DIC trends in the 2000s and 2010s

In addition to an increase in upwelling, stronger Westerlies in the Southern Hemisphere also lead to an increase in northward Ekman transport^11^, which at the surface brings sea ice and colder and fresher water from the Antarctic coast. Indeed, decreasing sea surface temperatures^33–35^ (cf. Fig. 5a) and increasing freshwater fluxes due to northward sea-ice transport and increased precipitation^36^ have been observed over the South Pacific sector starting in the 2000s. To understand the circulation in the Pacific and its role in transport of DIC, we consider effects on water-mass classes of specific neutral density (*γ*_*n*_) ranges: Circumpolar Deep Water (CDW, *γ*_*n*_ = 27.5−28 kg/m^3^), Antarctic Intermediate Water (AAIW, *γ*_*n*_ = 27.0−27.5 kg/m^3^), and Subantarctic Mode Water (SAMW, *γ*_*n*_ = 26.6 − 27.0 kg/m^3^)^37^. CDW comprises old, dense waters that upwell to the surface south of 55^∘^S; in the South Atlantic, this water-mass is North Atlantic Deep Water. AAIW comprises cold and fresh waters that travel northward from the upwelling zone and eventually sink to about 1 km depth, and SAMW of upwelled waters that continue to travel equatorward at the surface before sinking^25^ (cf. isocontours in Fig. 3).

A water-mass can gain buoyancy (become lighter) due to ice melt or lose buoyancy (become denser) due to brine rejection at the surface. In mid-2000s, an increase in melting of advected ice contributed to buoyancy gain of SAMW within the upper 700 m^37^, which was made even lighter by surface heating north of 40^∘^S^33^. Increased freshwater flux from ice melt also has made AAIW lighter, counteracting the buoyancy loss due to cooling at the surface^37,38^. In contrast, salt fluxes due to brine rejection led to buoyancy loss of CDW, but with large zonal differences. In the Atlantic sector (Weddell Sea), destruction of water-masses in the 27.6−27.8 kg/m^3^ neutral density range near the surface^37^ required water in this density range to upwell from the interior. However, in the Pacific sector (Ross Sea), positive formation rates of this density range near the surface^37^ weakened the upwelling.

These water-mass transformations can help explain the DIC trends in the Pacific that we find in the 2000s. Weakening of CDW upwelling south of 60^∘^S resulted in decreased delivery of old DIC-rich waters to the surface, and hence a weaker increasing trend in DIC near the surface in 2000s (Figs. 2b and 3e). In the 2010s, the near-surface DIC trends further decreased and became negative (Figs. 2c and 3f), while DIC built up (increasing trend) below 1 km depth at the latitudes of CDW upwelling (Figs. 2f and 3f). The decreasing DIC trends follow the AAIW and SAMW density isosurfaces northward, further pointing to weakened upwelling being responsible, as the upwelled CDW comprises a large portion of AAIW and SAMW.

Importantly, in addition to buoyancy gain of CDW, buoyancy loss (through cooling) of poleward-flowing subtropical surface waters contributes significantly to formation of SAMW^39–41^. These surface waters (*γ*_*n*_ < 26.6 kg/m^3^) are characterized by lower DIC concentrations than CDW, which is sourced from deeper ocean layers (cf. Fig. 1). Previous studies showed that intensification of the Southern Westerlies lead to increased heat loss and decreased freshwater input at the surface, resulting in increased SAMW formation rates^42^ and deepening of SAMW layer^43^. As such, negative trends in the upper portion of the Pacific sector could also be due to a proportional increase in contribution to SAMW formation from cooling of subtropical low-DIC waters rather than freshening of high-DIC CDW waters. Climatologically, these findings are important because a decrease in near-surface DIC concentrations can enhance the uptake of atmospheric carbon by the ocean. These trends correspond to the ocean pCO_2_ decreasing relative to the atmospheric pCO_2_ in the 2000s (cf. Fig. 5e, which suggests an increase in ocean carbon uptake potential.

However, recent satellite measurements^35^ found increasing sea surface temperatures over much of the Pacific sector in the 2010s (cf. Fig. 5c, l). Although the Westerlies also have weakened over the Pacific sector in the 2010s (cf. Fig. 5i) so upwelling would be suppressed, we find that the DIC trends from the 2000s have reversed in the 2010s and are predominantly positive in the Pacific. This reversal suggests that buoyancy forcing may play a relatively more important role than wind forcing in setting the DIC concentrations in the South Pacific, similar to the previously suggested thermally-driven trend pCO_2_ in the Pacific^11^.

Unlike the Pacific, most of the Atlantic and Indian sectors of the Southern Ocean, especially between 30 and 60^∘^S have been warming and storing heat in the upper 2 km over 1990s and 2000s^44,45^ (cf. Fig. 5a, b). The larger heat uptake over the Southern Ocean compared with the northern temperate and high-latitudes is partially because of the reinforcement of greenhouse gas-induced heating by ozone-hole forcing^46^ and low levels of aerosols, which could have a cooling effect^45^, in the Southern Hemisphere. Warming of the upper ocean stabilizes the water column, weakening the effects of the wind-driven upwelling around 50−55^∘^S. In the Atlantic sector, these changes are reflected in decreasing DIC concentrations along the upwelling density isosurfaces in 1990s and 2000s (Fig. 3a, b). Trends are also negative between 45 and 60^∘^S in the 2010s subsurface along the upwelling density isosurfaces, even though there is cooling at the sea surface (cf. Fig. 5c, l), suggesting that the trends could be due to the SAMW/AAIW zonally advected from the Pacific sector (Figs. 2c and 3c). In the Indian sector, we find similar negative trends south of 50^∘^S, but positive trends near the surface to the north (Figs. 2c and 3h, i). The regions of near-surface positive trends correspond to areas, where strong SAMW and AAIW formation rates^41,43,47^ are enhanced by salinity fluxes^48^ and increased Ekman pumping^43^, helping export DIC into the interior (Fig. 3b, c).

Furthermore, Atlantic Meridional Overturning Circulation (AMOC) has been weakening since the 1990s^49–51^. AMOC transports dense water sinking in the North Atlantic to the upwelling region in the South Atlantic. The slowdown of AMOC has been attributed to increased uptake of heat by the North Atlantic in response to rising atmospheric greenhouse gas levels^49^ and weakening of North Atlantic Oscillation since the early 1990s^45,52^. As a result, meridional transport has weakened and due to buoyancy gain, surface waters in the North Atlantic have been sinking to shallower depths, where DIC content is lower. These changes in the circulation dynamics, which diminish the connectivity between the ocean interior and surface layers, are consistent with our results: progressively decreasing trends along the upwelling density isosurfaces from the 1990s to the 2000s. Notably, in the 2010s, the decreasing trend in the Atlantic strengthens in the subsurface (cf. Fig. 3c) compared with 2000s, whereas near the surface DIC concentrations increase (cf. Fig. 2c) consistent with the decrease in ocean carbon uptake potential in the Atlantic (cf. pCO_2_ trends in Fig. 5f). Since the 2010s, increased AMOC transport has been recorded in the subtropics in the Northern Hemisphere^52,53^. However, because of the long temporal scales in ocean circulation, there will be a lag in response of the Southern Ocean upwelling and DIC concentrations to such changes in the North Atlantic.

## Discussion

Our results show some decreasing trend in DIC concentrations in the Southern Ocean over the period from 1993 to 2010, in particular in the Pacific sector. This trend is congruent with the previous findings of decreasing CO_2_ uptake in this region in the 1990s and increasing uptake in the 2000s^11,54^, and indicate the continuation of the increasing uptake potential at the ocean surface into the 2010s. Our findings are also in line with previous works on ocean uptake of anthropogenic carbon for the 1990s and 2000s^15,55^. While the upper layers of the Southern Ocean continued to uptake anthropogenic carbon, carbon accumulation rates have been lower than predicted based on the increase in anthropogenic CO_2_ in the atmosphere^15^. Furthermore, previous analysis^55^ showed negative trends in total and natural DIC in the upper Southern Ocean, similar to our findings, despite an increase in anthropogenic DIC. As such, previous studies attribute changes in DIC concentrations primarily to changes in ocean circulation^15,40^, which we address through the lens of watermass transformation in our study.

The overall increasing DIC trends in the 2010s that we find are qualitatively consistent with the results from a recent study^16^, which computed the decadal changes by comparing the spatially-interpolated data only from biogeochemical floats over the 2014−2019 period with shipboard measurements prior to 2005. Comparing with the DIC trends in the previous decades, it is possible that the Southern Ocean took up more carbon at the surface in the 2010s, thus increasing DIC near the surface, because it was undersaturated in carbon in the previous decade. Importantly, we find subsurface decreasing trends in DIC in the 2010s, in particular in the Atlantic sector, that are only weakly present in this previous study. Floats can augment shipboard data, in particular because of superior wintertime coverage. As carbon uptake in the Southern Ocean has strong seasonal signature^7,8,56^, in part due to biological activity, shipboard measurements, which are predominantly taken in the summer, may be affected by small scale processes that drive local primary production rates to be spatially and temporally variable. Furthermore, it has been found that models using only data from floats produce Southern Ocean carbon uptake values that are one-third of those from models using only using shipboard data^22^. As such, combining both shipboard and float measurements in models provides more accurate estimates of carbon flux and carbon concentrations^22^. Considering such differences between shipboard-only and float-only estimates, we integrated data from both shipboard and Argo float measurements into our model to make the estimations of DIC concentrations more robust.

Our results demonstrate that there are long-term (possibly decadal) changes in ocean DIC concentrations and thus carbon uptake. We find similar effects of weakening upwelling and connectivity between the deep and surface waters, which possibly inhibit export of carbon from the surface into the ocean interior, in different sectors of the Southern Ocean. Although these trends are in line with the expected changes in ocean circulation, what drives these changes varies zonally. The difference in the underlying mechanisms implies that responses to future changes in the circulation dynamics may also not be zonally uniform. In the current model, we are unable to separate changes in DIC concentrations due to uptake of anthropogenic carbon and due to natural variability in the ocean circulation; it may be pertinent to include methods from previous studies^15,16^ into future analysis. Here, we found a period of the decrease in DIC concentrations near the surface, which allowed for increased uptake of carbon from the atmosphere, followed by a period of increase in near-surface DIC concentrations, possibly due to weakened export into the interior. Continued monitoring efforts are necessary to assess the long-term impacts of DIC accumulation on storage of anthropogenic CO_2_ in the deep ocean. These changes are important not only from a climatological point of view, but also for the management of marine ecosystems, which are sensitive to acidification^57^. The model presented here can serve as a useful tool for such future studies as it is able to estimate DIC concentrations in the ocean interior up to 4 km depth from new satellite measurements as they become available.

## Methods

### Overview

In this study, we train a deep-learning model that finds non-linear relationships between the input variables (physical and biogeochemical parameters) and ocean DIC concentrations. The model is trained over a three-dimensional domain over the Southern Ocean confined latitudinally between 30^∘^S and 80^∘^S and vertically between the ocean surface and 4 km depth. Model training is conducted in two phases. In Phase 1, the model is trained using the three-dimensional distribution of DIC concentrations (available at least up to 4 km depth) from the output of B-SOSE (ocean circulation model). Phase 1 is necessary because B-SOSE output provides a large volume of data for model training, especially below 2 km, where observational measurements are sparser. In Phase 2, the model is trained further with DIC concentrations from shipboard measurements (available at least up to 4 km depth) and Argo float measurements (available up to 2 km depth). Phase 2 training is necessary to correct any biases from the B-SOSE model by incorporating real ocean measurements. One of the main advantages of our model is that it uses surface physical and biogeochemical data that is readily available from satellites as input variables. Hence, once the model is trained (using DIC measurements both at the surface and in the ocean interior), it can then be applied to new satellite data to estimate Southern Ocean DIC faster and in a less computationally expensive manner than other models (e.g., ocean circulation models or interpolated models).

### Deep-learning model

**Fig. 6:**
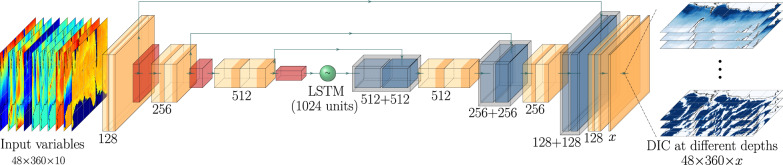
Schematic diagram of the U-net model. The green circle with a tilde in the middle denotes the long-short-term memory (LSTM) cell with 1024 units, which connects the encoder (the 9 layers on the left hand side) with the decoder (the 13 layers on the right hand side). The 3 × 3 convolutional layers are in light orange followed by the ReLU activations in dark orange. The 2 × 2 max pooling layers are in red. Light blue layers are the 2 × 2 up-convolutional layers, which are concatenated (shown as the gray boxes) with the forwarded features (shown as the dark blue layers) from the encoder. The arrows denote the residual learning connections that forward from the encoder to the decoder. To improve computational efficiency, *x* vertical layers are trained simultaneously. *x* = 2 from the ocean surface to 2 km depth and *x* = 3 for 2–4 km.

Our deep-learning model^58^ is a type of neural network that we adapted from the U-net model introduced in a previous study aimed to predict atmospheric ozone concentrations^17^. Similar architectures are also applied in other earth science studies^59^. The schematic diagram of the U-net model is shown in Fig. 6. The model consists of both convolutional neural networks and recurrent neural networks^60^. The first three convolutional blocks are used as an “encoder” to extract the hidden features about the spatial patterns in the input data and condense their information into so-called latent vectors. Each convolutional block consists of two convolutional layers and one max pooling layer. Outputs from the convolutional layers are activated by the Rectified Linear Unit (ReLU) function to enhance non-linearity of the deep-learning model. The trainable parameters in each convolutional layer are the convolutional filters in the convolutional layers. The output from the third convolutional block is then forwarded into a long short-term memory^61^ (LSTM) cell with 1024 units to capture the temporal dynamics in the latent vectors. After the LSTM cell, the latent vectors are projected back onto the DIC fields by a “decoder", which contains three up-convolutional blocks with descending depths. Similar to the encoding process, the up-convolutional layers are also activated by the ReLU function. Three residual learning connections are added from the encoder to the decoder, in order to stabilize the training^62^. The convolutional layers are all using convolutional filters with 3 × 3 size. The up-convolutional layers are using 2 × 2 filters. The max-pooling layers are also 2 × 2. We used the mean squared error loss function to train the deep-learning model on a NVIDIA T4 Tensor graphics processing unit (GPU). We applied the ADAM optimization algorithm to boost the speed of training^63^.

In this U-net model, we used sea surface temperature, sea surface height anomalies, ocean surface velocities, 10 m wind speeds, total heat flux at the ocean surface, ocean surface chlorophyll-a, and ocean surface partial pressure of CO_2_ (pCO_2_) as the input variables (predictors). The U-net model predicts DIC concentrations south of 30^∘^S in the upper 4 km of the ocean. These input variables attempted to capture physical (e.g., ocean circulation and mixing), biological (e.g., uptake of CO_2_ by photosynthetic organisms), and chemical (e.g., uptake of atmospheric CO_2_ at ocean surface) processes that may affect DIC distribution. While there are many other factors (e.g., sinking rates of organic matter, organic matter remineralization rates, total alkalinity, calcification) that could change DIC concentrations, we chose variables that could be easily measured at the ocean surface, as these measurements were better constrained and available at higher spatial and temporal resolutions than measurements in the ocean interior. We trained the U-net model to capture the relationship between surface predictors and DIC fields at different depths. In total, we trained 22 U-net models to cover the 48 vertical levels from ocean surface to the 4 km depth. We conducted the training of each U-net model in two phases: first augmenting the volume of data using a biogeochemical ocean circulation model, and then correcting for biases of this model using observational data. We detail the datasets that we used in  each of the training phases in the following sections.

Ocean carbon sink has been previously estimated using different methods. However, these methods may either produce indirect bulk estimates over an entire ocean basin (i.e., inverse models^64^), be numerically expensive (i.e., ocean circulation models^14^), or have limited temporal coverage (i.e., interpolations of direct measurements^15,16^). Our deep-learning approach attempted to address these issues. In our model, because of the high spatio-temporal availability of the satellite-based input variables, we were able to create a dataset of DIC concentrations at 1^∘^ horizontal resolution in the upper 4 km of the ocean at 5-day intervals between 1993 and 2019. It allowed us to create a timeseries and compute DIC trends at each individual grid cell over this time period. As a result, we were able to explore spatial patterns in temporal trends, rather than only comparing aggregate decadal averages as in previous studies^15,16^. Using neural networks is also advantageous, as they can capture non-linear relationships between the predictor variables, in contrast to the linear regression models used in previous studies^15^. In addition, this deep-learning model can compute DIC concentrations over the entire Southern Ocean domain very quickly, i.e., on the order of 1−2 T4 GPU computational hours required for one year of DIC calculations, which makes it ideal for future monitoring of the ocean carbon sink using new satellite data as it becomes available. Finally, it is important to note that a previous study^22^ showed that errors of neural network predictions are reduced when the domain is constrained to a single basin rather than the global ocean, and our model was developed and trained specifically over the Southern Ocean basin only.

### Data sets

B-SOSE^14^ is a data-assimilating model that incorporates Biogeochemistry with Light, Iron, Nutrients, and Gases model^65^ into a data-constrained general circulation model of the Southern Ocean (SOSE)^66^. The model has uniform horizontal resolution of 1/3^∘^ over 30−78^∘^S; spacing of 52 vertical layers varies with depth from 4.2 m near the surface to 400 m in the deepest layers. The output data contains both physical (e.g., temperature, salinity, flow velocity) and biogeochemical (e.g., concentrations of DIC, dissolved oxygen, pH, and chlorophyll *a*). It is available at 3-day intervals over the 2008−2012 period. The biogeochemical portion of the model includes carbon, nitrogen, and phosphorus cycling, phytoplankton population dynamics, and iron chemistry. The model assimilates in-situ observational data of the carbon system, oxygen, and nutrients from bgc-Argo, GLODAPv2^19^, and Surface Ocean CO_2_ product version 4 (SOCATv4)^67^ in addition to physical constraints from hydrographic and satellite observations.

We used data from the following sets produced based on satellite observations. All data were available between 1993 and 2019 over the Southern Ocean (i.e., south of 30^∘^S), with the exception of chlorophyll *a* (chl-a), which was only available north of 60^∘^S between 1993 and 1997. Horizontal ocean surface velocities (*u*, *v*) were obtained from Ocean Surface Current Analysis Real-time (OSCAR)^68^, which uses satellite sea surface height, wind, and temperature for computations^69^. Data are available at 1/3^∘^ and 5-day resolutions between 1992 and 2020. Sea surface height (SSH) was obtained from Copernicus Marine Environment Monitoring Service (CMEMS) dataset^70^ that merges altimetry data from available missions for a more consistent and homogeneous product. It is available at 1/4^∘^ and 5-day resolution between 1993 and 2020. SSH was used to compute vertical velocity (w) at the ocean surface to be consistent with calculations in B-SOSE. Zonal and meridional components of 10 m wind speed, sea surface temperature (SST), and total heat flux at the ocean surface were obtained from ERA5^71^, which is a comprehensive reanalysis dataset that assimilates available observations in the upper air and near surface. Data^72^ are available at an hourly temporal resolution and 31 km spatial resolution from 1979 to 2020. Total heat flux was computed as the sum of net shortwave and longwave radiation and sensible and latent heat, using the hourly accumulation values (in J/m^2^) converted to flux units (W/m^2^). Surface chl-a concentrations were obtained from GlobColour dataset^73^ by the European Space Agency, which merges data from four satellite sources. Data used here is available at 1/4^∘^ and 8-day resolution from 1997 to 2020. An estimate from neural network^18^ was used for pCO_2_. This neural network uses primarily satellite observations as inputs to interpolate the available shipboard measurements of pCO_2_ over 1^∘^ grid at a monthly resolution from 1982 to 2020. Using this neural network-based dataset is advantageous compared to simply spatially-interpolated observations because it accounts for spatial and temporal heterogeneity of observational data availability.

We trained the model with DIC data from two observational datasets. The first one was GLODAPv2^19,20^, which is a compilation of inorganic carbon data collected during research cruises. We used in-situ data from the original shipboard measurements rather than a globally remapped product. The second dataset was collected by SOCCOM^21^ project Argo floats equipped with biogeochemical sensors. Here we only use data with “good” quality flag. We used GLODAPv2 shipboard measurements available between 1998 and 2019 and Argo float measurements available between 2014 and 2019. Over the period where the two datasets overlap, the number of Argo float measurements was much larger than that of the shipboard measurements (cf. Supplementary Fig. 1). Argo float data also had better temporal coverage, whereas wintertime shipboard measurements were limited^22^. However, data from Argo floats was only available above 2 km depth, whereas there were shipboard measurements below this depth, though far less numerous than above (cf. Supplementary Fig. 1). Furthermore, it has been shown using both Argo float and shipboard measurements in neural network training minimizes the root mean square error between the model predictions and observations^22^, so we used both datasets for training our model.

### Model training

The high spatial and temporal resolutions of B-SOSE over a three-dimensional domain made it a good training set for a deep-learning model. B-SOSE data was also more evenly distributed spatially and temporally than the observations. In particular, it had significantly more data points available below 2 km, where observations were especially sparse. Thus, including B-SOSE dataset into training was important to prevent overfitting of the deep-learning model to the observational data. To correct for any inherent errors of the B-SOSE model and to account for its short availability period (only 5 years), it was also necessary to further train a model with observed data (i.e., shipboard and Argo float measurements). However, because of the vast difference in the number of available data points between B-SOSE (~10 million data points per timestep over 609 timesteps) and observations (~450, 000 data points in total), it was necessary to train the model in two phases; otherwise, the deep-learning model output would have been heavily biased towards B-SOSE. Finally, because the near-coastal processes in shallow waters may be significantly different from the dynamics of the open ocean, we excluded regions with less than 1 km depth from our model training.

In the first training phase of the deep-learning model, we used SSH, ocean surface velocities (*u*, *v*, *w*), ocean surface heat flux, pCO_2_, and chl-a concentrations from B-SOSE output and SST and 10 m wind speed velocities from ERA5. We chose to use these two predictors from ERA5 rather than B-SOSE output because of the higher spatio-temporal resolution of the ERA5 data, which would be advantageous for matching to the in-situ measurements in Phase 2 of the model training. The hourly ERA5 data was averaged over 3-day period to have the same temporal resolution as B-SOSE. DIC concentrations from B-SOSE were taken as the target for model training. We randomly sampled 85% of the B-SOSE outputs over the 2008−2012 period for model training, while reserving a randomly-sampled 10% of it for in-sample validation to prevent overfitting. The remaining 15% of the data set was then used as out-of-sample validation set for the model.

The comparison with model-predicted DIC from Phase 1 training and B-SOSE DIC is shown in Supplementary Fig. 2 for the out-of-sample validation set averaged over 1 km depth intervals. The deep-learning model (middle) generally reproduced the B-SOSE DIC (left) for each depth interval. Errors (right) were mostly less than ±10 μmol/kg and patterns in error distribution did not show any apparent bias.

Box-plot of errors binned by 1 km depth intervals shows that the errors were centered and symmetrically distributed around approximately zero at all depths (cf. Supplementary Fig. 3a). The errors showed overall no systematic bias towards high or low values, and the errors were within ± 15 μmol/kg with the IQR less than ±5 μmol/kg. The spread was larger in the upper 1 km, possibly related to a greater degree of noise associated with small-scale near-surface processes that was more difficult to capture with the model. Horizontally-averaged profile of model-predicted DIC concentration also showed very small deviation (less than 2 μmol/kg deviation from B-SOSE data across different depth levels (cf. Supplementary Fig. 3c, d)).

The heatscatter plot of DIC concentrations predicted by the deep-learning model over the three-dimensional domain for 2012 is shown in Supplementary Fig. 3b in comparison with B-SOSE DIC concentrations. The vast majority of the points were along the one-to-one line with a high linear correlation coefficient (*r*^2^ = 0.97) between the model-predicted and B-SOSE DIC concentrations and relatively small RMSE of 5.4*μ*mol/kg.

In the second training phase, we transferred the U-net model weights obtained from Phase 1 to the satellite-based observational data described above and further trained the model to minimize the RMSE between the model predictions and shipboard and Argo float measurements. When chl-a measurements were not available (primarily due to presence of sea ice), values within those cells were set to zero to be consistent with B-SOSE instead of setting it to a non-zero minimum chl-a concentration value like in some previous pCO_2_ models^74^. The observational DIC data was re-mapped to the same depth levels as the B-SOSE dataset to be consistent with Phase 1 training output. We randomly sampled 20% of the observational data as an out-of-sample test dataset and used the remaining 80% as the training dataset. Again, a randomly-sampled 10% of the training set was used for in-sample testing. To compare the two observational DIC datasets, we trained the model with (1) only shipboard data, and (2) with shipboard and Argo float data.

The distributions of relative errors of the model prediction (cf. Supplementary Fig. 4a) were again mostly symmetric around zero. Spread of the errors is larger than in Phase 1 training, which could be the result of model prediction errors, the variability in data collection from different cruises, and any systematic differences between shipboard and Argo float measurements. As expected, the correlation between predicted and observed DIC concentration values improved when the model is trained with more data points by including the Argo float measurements (compare Supplementary Fig.  4a,c). When the model was trained with both shipboard and Argo float data, considerably more model-predicted points fell along the one-to-one line and RMSE improved. This result is consistent with previous analysis of neural networks used for to compute pCO_2_, concluding that both shipboard and Argo float data were necessary for more accurate model predictions^22^. However, because of the much more limited number of observations compared with the number of available B-SOSE data points, the linear fit (e.g., correlation coefficient) was worse compared with Phase 1 training (cf. Supplementary Fig. 3b) and RMSE is higher (~13 μmol/kg). This demonstrated that performance of a deep-learning model improved with more data points available for training and why it was important to pre-train the model with a large volume of B-SOSE data in Phase 1.

In order to further validate our results, we also compared annual DIC trends calculated using shipboard measurements with annual DIC trends calculated using our deep-learning model predictions in Phase 2. We grouped all available shipboard measurements by latitude, longitude, and depth (1^∘^ intervals and depth intervals corresponding to B-SOSE, which increase with depth) and found the mean DIC at each location for each time stamp. We then calculated linear trends in DIC concentrations for all locations where at least three temporal data points were available. Using our deep-learning model predictions at the same locations and times, we also calculated linear trends in model-predicted DIC concentrations. These trends for selected hydrographic transects are shown in Supplementary Fig. 5. The bottom panels show the ratio of shipboard-based DIC trends to model-based DIC trends. Overall, this ratio was positive, meaning that our model predicted DIC trends of the same sign (i.e., increasing or decreasing DIC concentrations) as the shipboard measurements. Supplementary Fig. 6 shows (a) the correlation between shipboard-based DIC trends and model-based DIC trends and (b) the ratio of the two DIC trends. The slope of 0.95 and *r*^2^ = 0.98 of the correlation suggest that our model predictions of DIC trends agreed well with the shipboard measurements. The ratio of the trends was also predominantly positive centered around 1, suggesting that the model-based trends were of similar value and sign as the shipboard-based trends. It is important to note that shipboard measurements are sparse and for each spatial grid point (latitude-longitude-depth), there are very few temporal values to compute trends. Hence, trends computed using so few values are subject to bias due to sampling timing (i.e., potentially sampling during unsual conditions). As a result, DIC trends shown here do not necessarily agree with the DIC trends shown in Figs. 2, 3, which were computed using monthly data obtained from the deep-learning model predictions, and therefore, typically an order of magnitude or more temporal data points.

### Linear decadal trend estimations

We applied the trained deep-learning model to the satellite-based observational datasets to compute DIC concentrations at 1^∘^ horizontal resolution and at the same vertical levels used in the B-SOSE model. DIC concentrations were computed at a 5-day resolution over the period between 1993 and 2019, for which the input variables were available. Chlorophyll-a concentrations were only available after 1997 and for the prior years, climatologically-averaged chl-a concentrations computed over 1997−2019 were used. Same technique was applied to a previous neural network predicting pCO_2_^11^. We then divided the obtained DIC concentration data into three approximately decadal time periods: 1993–1999, 2000–2009, and 2010–2019. This division was useful in comparing the evolution of linear trends across different sectors of the Southern Ocean and relating our results to the previous findings of a weakening trend of the Southern Ocean carbon sink in the 1990s^9,10^ and a strengthening trend in the 2000s^11,54^.

Timeseries over each decadal segment were then extracted at each (latitude, longitude, depth) grid cell. In order to fill in the missing data points in the timeseries, which could result from ice or cloud cover or other problems with observational data, we used cubic-spline interpolation. However, to prevent over-interpolation at a location where too much data was missing, we applied criteria used in a previous study for gap-filling ocean-carbon data^75^. Namely, we restricted the interpolations to locations where data was available (1) for at least five years over each decadal period to ensure that the timeseries was long enough to capture seasonal and long-term trends, and (2) for at least 2/3 of a year at some point in the timeseries in order to extract seasonal cycles. Once the missing data was filled according to these two criteria, we subtracted the seasonal cycle, which we calculated over each time period individually using the statsmodels statistical module^76^. Computing seasonal cycle over each decade rather than using a climatological seasonal mean better accounted for any changes in the seasonal cycles over time. Finally, at each grid cell, from the seasonally-detrended data, we computed linear trends over each decadal period using a linear regression model and excluded trends that are not statistically significant (i.e., outside of the 95% confidence level with *p* ≥ 0.05). The statistically significant linear trends were then used to produce Figs. 2 and 3 in the main text. The same technique for calculating linear temporal trends was applied to satellite sea-surface products. Annual trends for sea surface temperature, difference between ocean and atmosphere pCO_2_, near-surface zonal (west-to-east) wind speed, and net surface heat flux are shown for each time periods in Fig. 5. For (d–f) ΔpCO_2_, positive values indicate increase in ocean pCO_2_ compared with atmospheric pCO_2_, thus reduced ocean capacity for uptake of atmospheric carbon. For (j-l) changes in heat flux, positive indicates warming at the sea surface (heat into the ocean).

## Supplementary information

Supplementary Information

## Data Availability

The dataset of DIC concentrations over 1993-2019 period computed by the deep-learning model presented in this study have been deposited in the Dataverse database under accession code 10.5683/SP2/FTQYTV.

## Code availability

Codes for Phase 1 and 2 training and testing of the model and for computing DIC from satellite-based products described in Methods can be found at 10.5281/zenodo.6569925.
