# Supplementary material for: A deep-learning estimate of the decadal trends in the Southern Ocean carbon storage
Source: Nat Commun. 2022 Jul 13;13:4056. doi: 10.1038/s41467-022-31560-5 (PMC9279406; doi:10.1038/s41467-022-31560-5)
Supplement: Supplementary file 1 — Supplementary Information [file 41467_2022_31560_MOESM1_ESM.pdf]

A deep-learning estimate of the decadal trends in the Southern Ocean carbon storage

Varvara E. Zemskova*[1], Tai-Long He*[1], Zirui Wan[1], and Nicolas Grisouard[1]

[1]Department of Physics, University of Toronto, Toronto, ON, Canada

Corresponding Authors: Dr. Varvara Zemskova (barbara.zemskova@utoronto.ca), Tai-Long He (tailong.he@mail.utoronto.ca)

Supplementary Figures

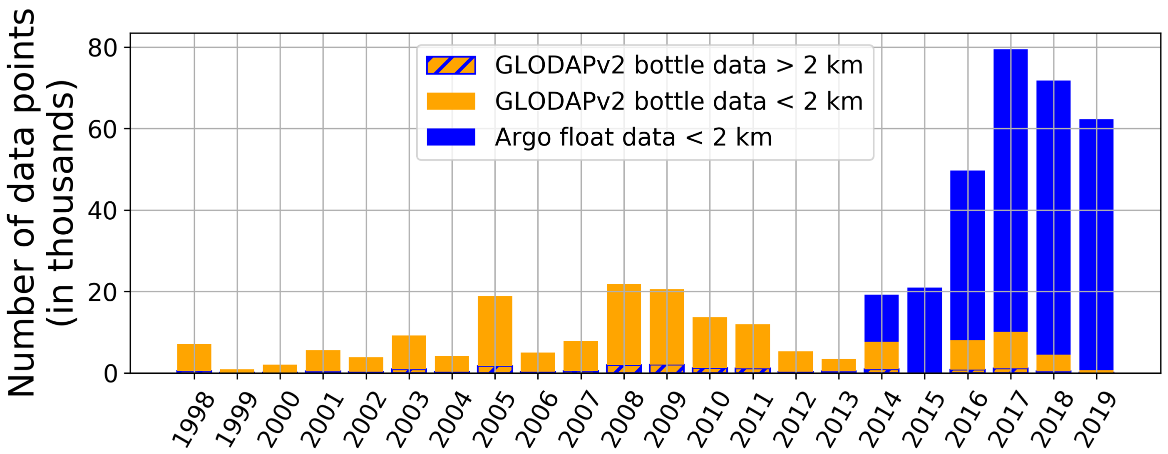

Supplementary Figure 1: Number of interpolated in-situ data points in each year between 1998 and 2019. Shipboard (GLODAPv2) data is available up to 4 km depth, and Argo float data is available only above 2 km depth.

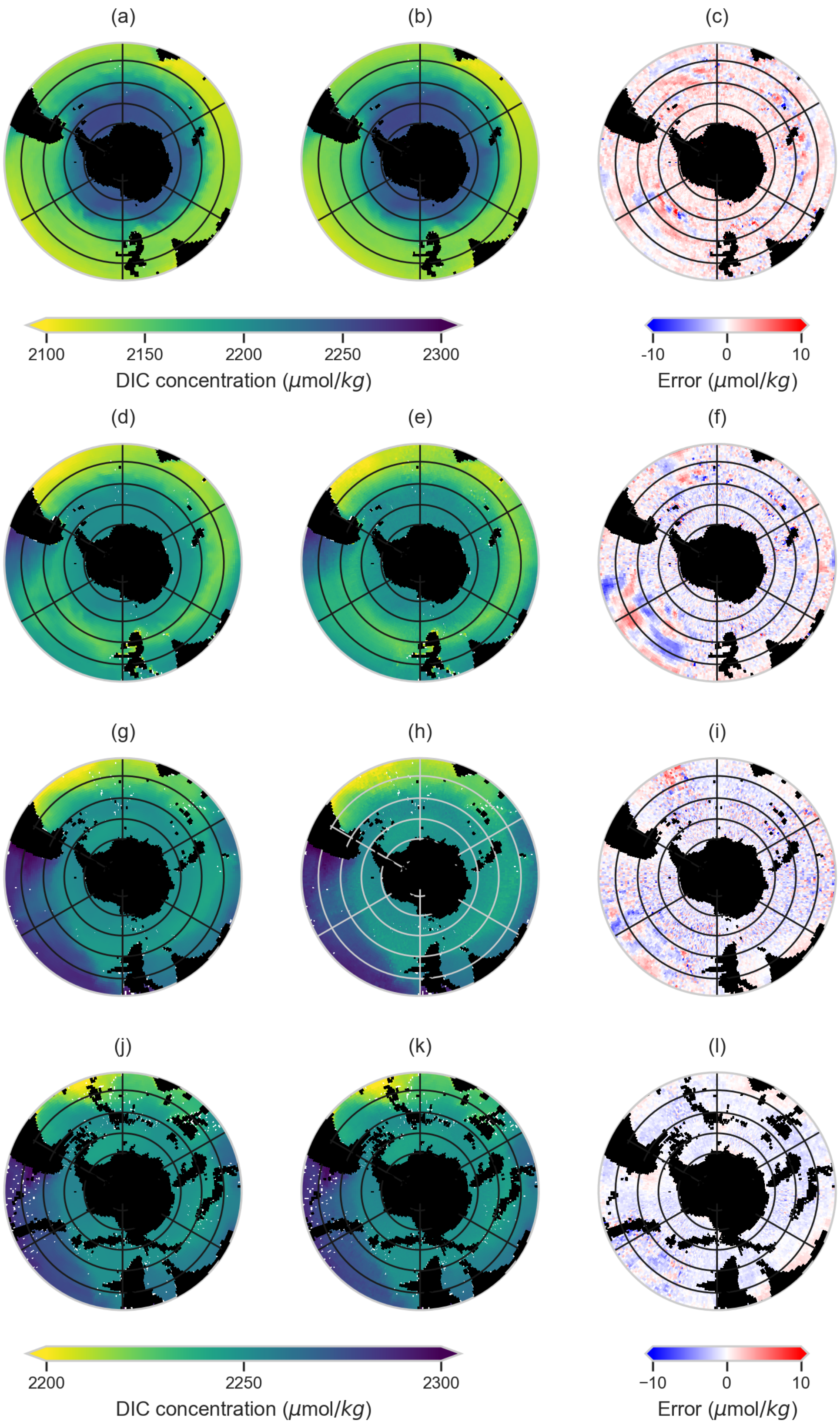

Supplementary Figure 2: Dissolved inorganic carbon (DIC) concentration vertically averaged over different depth intervals. (a, d, g, j) B-SOSE DIC concentration from B-SOSE averaged over, (b, e, h, k) DIC concentration predicted by the deep learning model, and (c, f, i, l) absolute errors of the deep learning model predictions. All variables are averaged over (a-c) 0-1 km, (d-f) 1-2 km, (g-i) 2-3 km, and (j-l) 3-4 km. Figures were made with Natural Earth. Free vector and raster map data (`naturalearthdata.com`) using Cartopy[1].

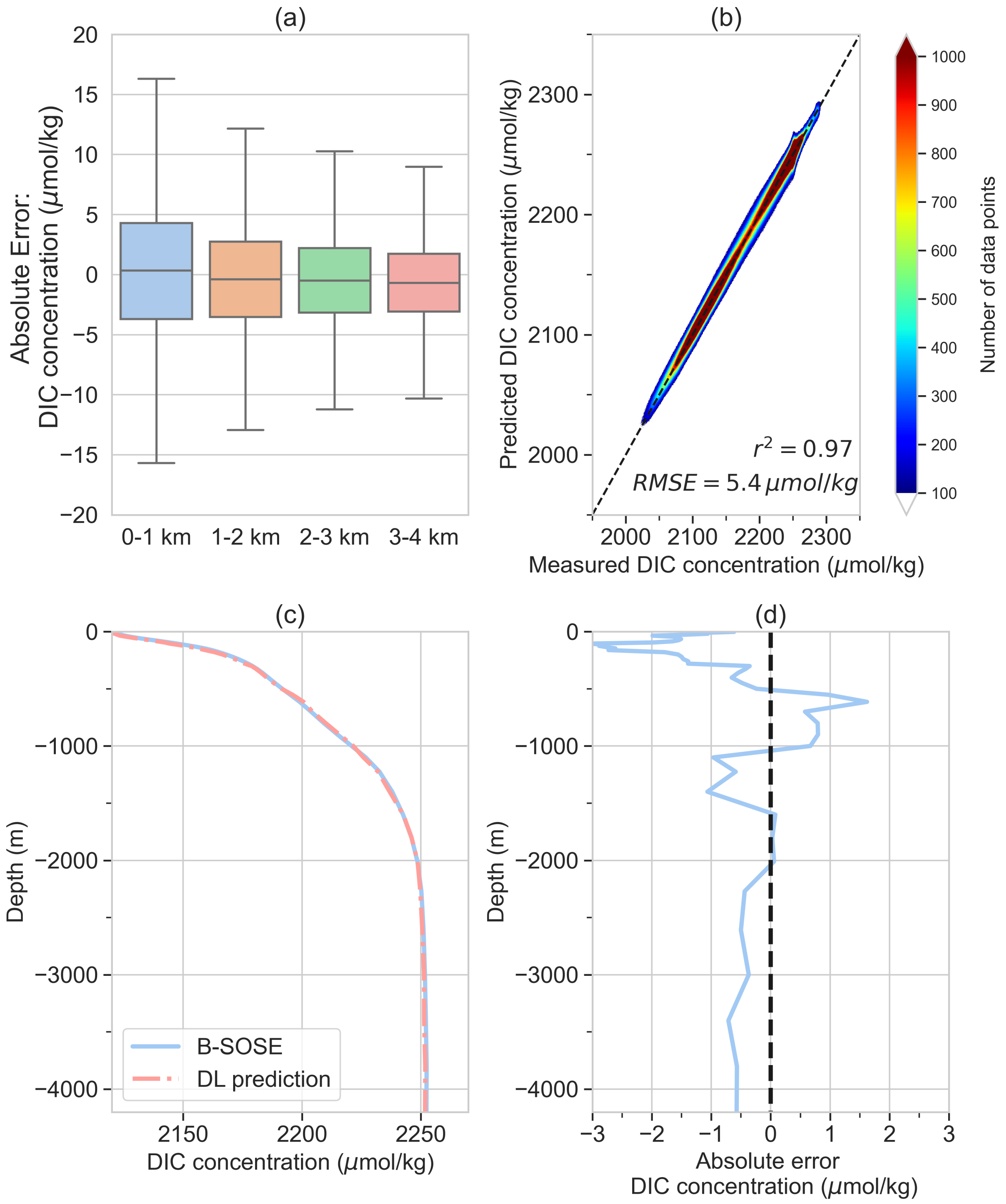

Supplementary Figure 3: Phase 1 training errors between deep learning model predictions and B-SOSE dissolved inorganic carbon (DIC) concentration. (a) box-plot of errors calculated for four depth intervals, (b) correlation between B-SOSE and deep learning model predicted DIC concentrations with linear fit r^2 and root-mean-square error (RMSE) shown, (c) horizontally-averaged DIC concentration for deep learning and B-SOSE model, (d) horizontally-averaged errors with depths. Differences between the model predictions and B-SOSE DIC concentration are calculated at each B-SOSE grid point, averaged over the test period (year 2012). In box-plots, center line: median; box limits: upper and lower quartiles; whiskers: $1.5\times$ interquartile range; points: outliers.

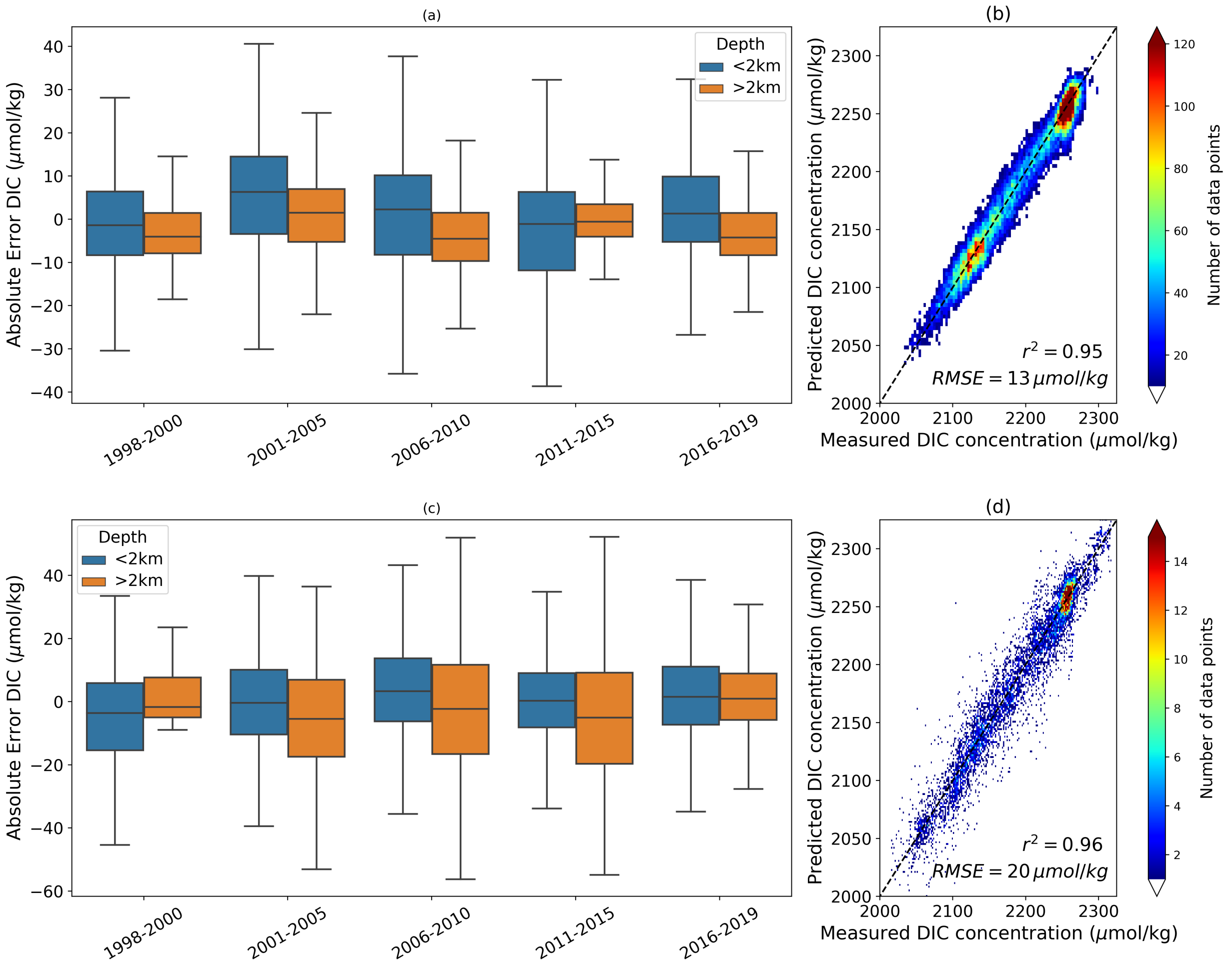

Supplementary Figure 4: Errors for the Phase 2 predicted dissolved inorganic carbon (DIC) concentrations compared to the measured DIC. (a) Paired box-plots for errors binned into the specified time intervals. For each time interval, left box-plot is for measurements above 2 km depth and right box-plot for measurements below 2 km depth. In box-plots, center line: median; box limits: upper and lower quartiles; whiskers: 1.5× interquartile range. (b) Correlation between DIC predicted by the deep learning model and measured DIC. One-to-one line in plotted in dotted black along with the regression coefficient r^2. (c, d) Same as (a, b) but for Phase 2 model trained only with GLODAPv2 shipboard measurements.

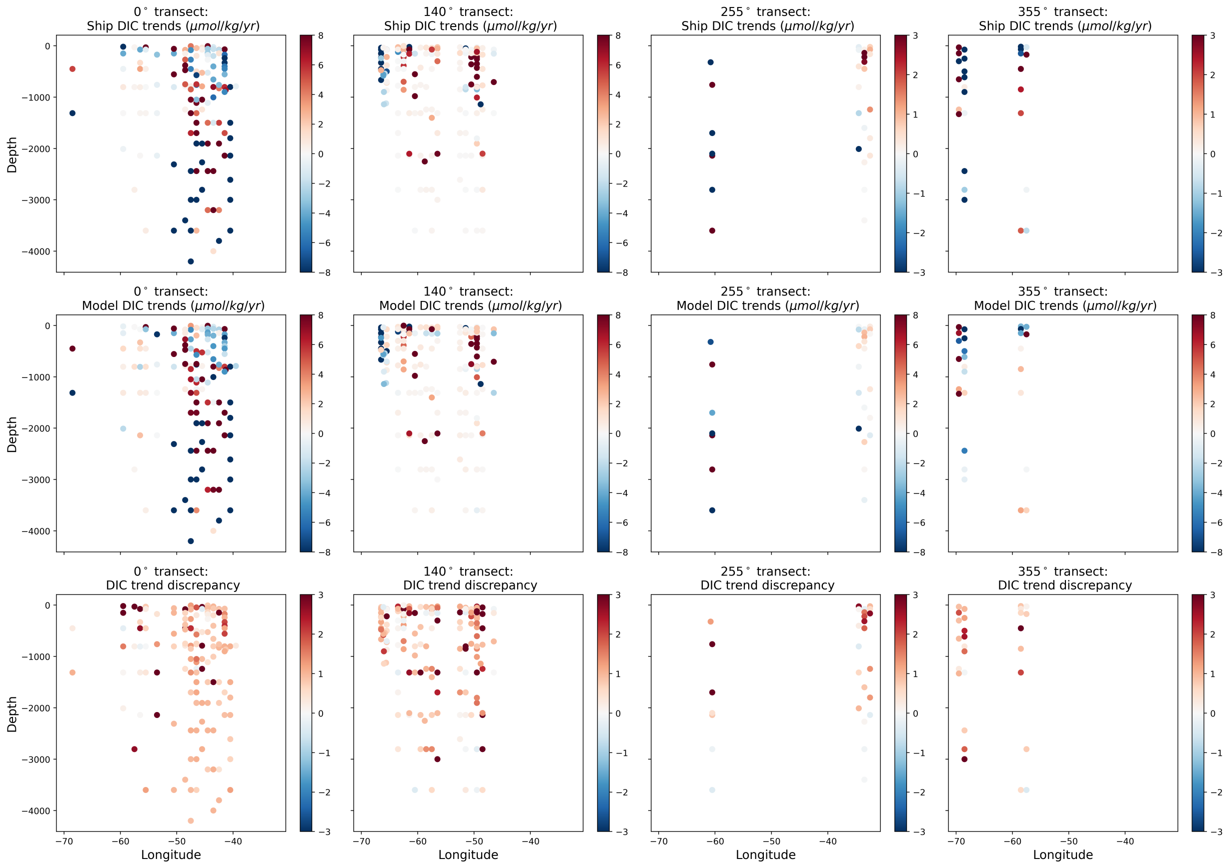

Supplementary Figure 5: Annual dissolved inorganic carbon (DIC) trends computed at four selected repeated ship transects. (top) trends calculated using shipboard data, (middle) trends calculated using deep learning model predictions using the same spatio-temporal points as the shipboard data, (bottom) ratio of DIC trends using shipboard measurements to DIC trends using model predictions. Positive (negative) ratio indicates that model predicts DIC trends of the same (opposite) sign (i.e., increasing or decreasing trends) as the shipboard measurements.

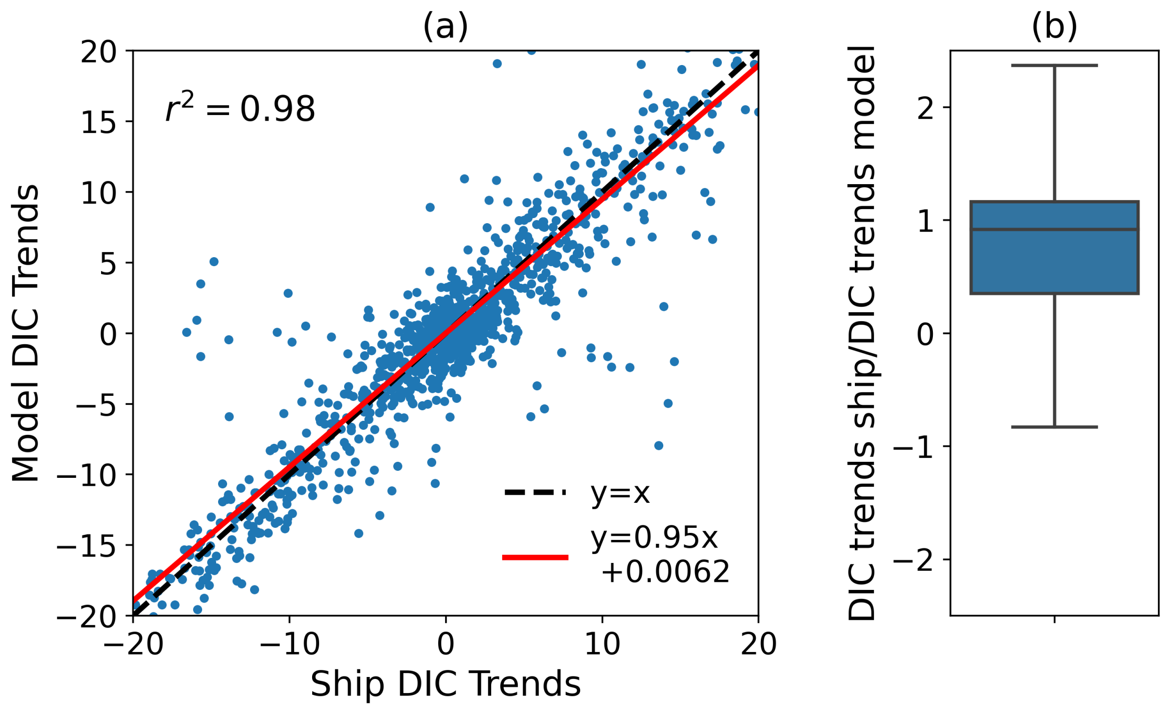

Supplementary Figure 6: Comparison of annual dissolved inorganic carbon (DIC) trends computed from repeated shipboard measurements with those computed from deep learning model predictions. (a) Correlation between the shipboard-based and model-based trends. (b) Box-plot of the ratio of DIC trends using shipboard measurements to DIC trends using model predictions. Positive (negative) ratio indicates that model predicts DIC trends of the same (opposite) sign (i.e., increasing or decreasing trends) as the shipboard measurements. Model trends are computed using the same spatio-temporal points as the shipboard measurements.

Supplementary References

[1] Met Office. *Cartopy: a cartographic python library with a Matplotlib interface*. Exeter, Devon, 2010 - 2015. URL: `https://scitools.org.uk/cartopy`.
